# Rehabilitation training improves exercise tolerance after percutaneous coronary intervention

**DOI:** 10.7555/JBR.26.20110119

**Published:** 2012-05-29

**Authors:** Fang Cui, Yusheng Ren, Heng Jin, Bo Cui

**Affiliations:** a Department of Rehabilitation Medicine, Dongfang Hospital, Tongji University, Shanghai 200120, China;; b Department of Cardiology, Shanghai Changzheng Hospital, Second Military Medical University, Shanghai 200003, China;; c Department of Cardiovascular Medicine, the Second Affiliated Hospital, Nantong University, Nantong, Jiangsu 226001, China; d Departments of Surgery and Pathology, Duke University Medical Center, Durham, NC27710, USA

**Keywords:** coronary heart disease, percutaneous coronary intervention, rehabilitation training, exercise tolerance, treadmill exercise test

## Abstract

The aim of this present study was to investigate the effects of training on exercise tolerance of patients with coronary heart disease after percutaneous coronary intervention. Fifty-seven cases of coronary heart disease after percutaneous coronary intervention were divided randomly into the rehabilitation training group (26 cases) and control group (31 cases). Patients in the rehabilitation training group received rehabilitation training at different stages and exercise intensities 3 d after percutaneous coronary intervention for 3 months. The heart rate, blood pressure, ECG changes in treadmill exercise test, and the frequency of anginal episodes were observed. The results showed that NST and ΣST of ECG and the frequency of anginal episodes were significantly reduced in the rehabilitation training group. In addition, exercise tolerance was improved and the total exercise time was lengthened in these patients. Moreover, ST segment depression time and emergence time of angina with exercise were also lengthened compared with controls (*P* < 0.05, or 0.01). However, the heart rate and blood pressure before and after exercise of the two groups were similar. The study indicated that rehabilitation training could significantly relieve angina, amend ischemic features of ECG, and improve exercise tolerance of coronary heart disease patients after percutaneous coronary intervention.

## INTROCUTION

Percutaneous coronary intervention (PCI) is a safe, effective, non-surgical treatment for coronary artery disease[1]–[3]. There has been an exponential increase in the demand for PCI since the 1980s, which has become the treatment of choice for many individuals with coronary artery disease. As a consequence, more and more people are now living with the disease. Since PCI is not a cure, strategies aiming at lessening disease progression and improving the cardiac functional status in these individuals are critical to an improved therapeutic outcome. Exercise is a central component of any cardiac prevention and rehabilitation strategy. Individualized exercise programs that incorporate physical activity and counseling can potentially improve the clinical outcomes of these patients[4]–[6]. Epidemiological data indicate that exercise and risk factor control can improve the quality of life in patients with coronary heart disease, reduce the incidence of coronary heart disease in the population and mortality[7],[8]. Regular high-intensity interval exercise training was found to significantly reduce late luminal loss in the stented coronary segment, which was associated with increased aerobic capacity[9],[10]. However, further larger studies are needed to fully investigate the effects of exercise in patients with PCI. In this study, we studied the effect of exercise on exercise tolerance in 57 patients with coronary heart disease after PCI by measuring the exercise tolerance in the treadmill test, heart rate changes, ECG changes and onset of anginal episodes before and after exercise training.

## SUBJECTS AND METHODS

### Subjects

A total of 57 patients were diagnosed and underwent successful PCI. Coronary heart disease was diagnosed by the WHO coronary heart disease diagnostic standard. The subjects were randomized into the rehabilitation training group and control group. The exclusion criteria included those patients with myocarditis, cardiomyopathy, pulmonary heart disease, or glaucoma, and with liver and kidney abnormality. Patients were also excluded if they had severe dysrhythmia, complete left bundle branch block and other contraindications for treadmill test. Patients were also excluded if they had acute myocardial infarction or a second coronary artery reconstruction within the previous month. The study protocol was approved by the local institutional review board at the authors' institutions and informed consent was obtained from all study participants.

### Treatment

Apart from PCI, the two groups received identical conventional therapy including oral aspirin, nitrates, angiotensin I convertase inhibitors or statins. The rehabilitation training group began rehabilitation training 3 d after PCI and during each training session ECG was monitored. Rehabilitation training was carried out in 3 stages 1. In the ICU stage, patients walked slowly and each session lasted 5 to 10 min. The target heart rate (THR) was less than 50% of the limiting heart rate. 2. In the regular ward stage, patients carried out regular rhythmic low-intensity exercise. 1) The patients were asked to walk or slowly climbing or descending the stairs for 5 to 10 min per session and 3 to 4 sessions/d. The THR was 50% of the limiting heart rate. 2) The patients slowly exercised the upper or lower limb and did body stretch for 5 to 10 min/session and 3 to 4 sessions/d. The THR was 50% of the symptom-limiting heart rate. 3) In the pre-discharge stage, the patients were asked to walk on level ground for 3 to 5 min and gradually increased the pace of walking until the patient started to feel fatigue. Exercise intensity was set at the THR at 65% to 80% of the maximum heart rate. The exercise time was 20 to 40 min and the mode of exercise was bicycling, boat rowing, hand and arm swing car, and treadmill exercise. Relaxation exercise for 5 to 10 min after regular exercise could return blood pressure and heart rate to the pre-warm up level. The frequency of exercise was 2 to 3 sessions/week, which consisted of warm up period, exercise period, recovery period. If discomfort appeared during exercise, appropriate drugs were taken and risk control measures were carried out. In the meanwhile, periodic educational sessions on prevention and treatment of coronary heart disease, risk factor control and detection, dietary guideline for coronary heart disease and psychological counseling were provided. The control group did not receive rehabilitation training. After 3 months, treadmill ECG was carried out (GE Marqurtte 2000 treadmill, General Electric Co., Fairfield, CT, USA) using the modified Bruce method.

Exercise amount was the sub-maximum amount and the primary endpoints were 1) the occurrence of the maximum predicted heart rate; 2) the occurrence of typical angina; 3) a decrease of blood pressure ≥10 mmHg during exercise; 4) failure to continue exercise because of fatigue, or heart rate did not increase after exercise and fatigue, chest tightness or shortness of breath occurred; 5) the occurrence of frequent premature ventricular heart beat, ventricular tachycardia, atrial fibrillation, atrial tachycardia or other malignant dysrhythmia; 6) the patients met the positive standards, i.e. ischemic ECG changes after or during exercise: ST segment decline ≥1 mm, lasting more than >2 min. If angina occurred during the study, nitroglycerin was administered. Before and after treatment, liver and kidney function, blood lipid, glucose, blood uric acid, electrolytes, blood and urine routine chemistry, ECG at rest and treadmill test, heart rate, blood pressure, rate-pressure product at rest and during exercise were recorded. The following parameters were also measured: ST segment decline per the number of sites (NST), the sum of ST depressions (ΣST), total exercise time, the time from the start of exercise to a depression of 1 mm in ST segment, the time from the start of exercise to the onset of angina, maximum depression of ST segment, and maximum exercise tolerance (METs). The number of anginal episodes, nature of pain, and the number of times of nitroglycerin taken during treatment was recorded and heart rate, blood pressure and side effects were measured.

**Table 1:** Effect of rehabilitation training on the heart rate and blood pressure after percutaneous coronary intervention (PCI) in patients with coronary heart disease

Group	Number of cases	Atrest				During exercise			
		HR(beat/min)	SBP(kPa)	DBP(kPa)	RPP	HR(beat/min)	SBP(kPa)	DBP(kPa)	RRP product
Exercise group	26	75.28 ± 12.16	17.66 ± 2.43	8.78 ± 1.26	1328.21 ± 133.52	142.12 ± 13.26	21.57 ± 4.29	10.27 ± 1.38	3087.39±353.32
Control group	31	74.87 ± 13.32	17.54 ± 2.51	8.64 ± 1.35	1313.37 ± 14.67	144.31 ± 14.82	23.36±5.26	10.53 ± 1.86	3321.65 ± 417.71

Compared with the controls, *P < 0.05, ^#^*P* < 0.01. HR: heart rate; SBP: systolic blood pressure; DBP: diastolic blood pressure; RPP: rate-pressure product.

(*x*±*s*)

### Statistical analysis

Data were expressed as *x*±*s* and analyzed using the SPLM software (Department of Statistics, the Fourth Military Medical University, Xi'an, Shaanxi, China) and Student's *t* test and ANOVA were performed for difference between groups. A *P* value less than 0.05 indicated statistical difference.

## RESULTS

Fifty-seven patients were recruited in the study. They included 44 males and 13 females whose age ranged from 48 to 69 years with an average of 58.4±6.3 years. Twenty-one patients were randomized into the rehabilitation training group and 31 patients were randomized into the control group. The age for the two groups was 59.4±5.9 years and 58.3±6.1 years, respectively, with a male to female ratio of 21/5 and 23/8, respectively. They included 35 cases of stable angina, 17 cases of unstable angina, 5 cases of old myocardial infarct, 23 cases of combined primary hypertension, 12 cases of diabetes mellitus, and 19 cases of hyperlipidemia. No statistical difference in disease course, complications and clinical features was observed between the two groups. The number of anginal episodes during the follow-up period for the rehabilitation training group and control group was 4.84±1.62 and 5.13±2.07 episodes/week, respectively. The amount of nitroglycerin taken was 2.73±0.93 and 2.88±1.31 mg/week, respectively, and there was no statistical difference between the two groups.

Treadmill test revealed no statistical difference in heart rate, systolic and diastolic pressure, heart rate at rest between the rehabilitation training group and control group (*P* > 0.05). Rate-pressure product during exercise was lower in the rehabilitation training group than the control group, but no statistical difference was found (*P* > 0.05). Total exercise time 3 months after therapy for the rehabilitation training group was significantly longer than that of the controls, and MET was also markedly increased (*P* < 0.05). The time from the start of exercise to ST segment depression of 1 mm, and time from the start of exercise to the onset of angina in the rehabilitation training group was also significantly longer than that of the controls (*P* < 0.01). During peak exercise, ST segment depression was more noticeable in the rehabilitation training group than the controls with a statistically significant difference (*P* < 0.01). Angina during exercise occurred in 5 cases in the rehabilitation training group and 11 cases in controls and the difference between the two groups was statistically different (*P* < 0.05) (***Table 1*** and ***Table 2***).

Changes in liver and kidney function, blood lipid, blood sugar, blood uric acid, electrolytes and blood and urine routine chemistries were of no clinical significance. No other side effects were found.

**Table 2:** Effect of rehabilitation training on main parameters during treadmill test

Group	Number of cases	Total exercise time (min)	Time to ST depression 1 mm (min)	Maximum ST depression (mm)	Time to anginal episode (min)	Maximum exercise tolerance (METs)
Rehabilitation training group	26	7.64±2.31*	5.87±1.45^#^	1.32±0.37^#^	6.79±1.63^#^	6.85±1.64*
Control group	31	6.21±2.34	4.12±1.83	1.76±0.56	5.34±1.68	5.83±1.67

Compared with the controls, *P < 0.05, ^#^*P* < 0.01.

(*x*±*s*)

## DISCUSSION

Over recent years, with the development of cardiovascular medicine, rehabilitation for coronary heart disease has evolved into rehabilitation after myocardial infarction and into rehabilitation after interventional therapy[11]–[14]. It has been shown that rehabilitation training for patients after PCI could noticeably increase the physical and working capacity of patients with coronary heart disease, improve blood supply to the ischemic myocardium. In addition, regular aerobic exercise and appropriate dietary control have been shown to lower triglycerides and increase the high density lipoprotein cholesterol ratio (HDL-C/TC)[15],[16]. These findings suggest that rehabilitation training helps delay or prevent atherosclerosis of the coronary arty and improve cardiac functional status after PCI. In addition, prescribed rehabilitation exercise has become one of the methods for management of cardiovascular diseases.

We studied the effect of rehabilitation training on exercise tolerance in patients with coronary heart disease who received PCI by using treadmill exercise test and we found no difference in heart rate, systolic pressure and diastolic pressure at rest and during exercise. We also found no difference in heart rate and rate-pressure product at rest between the two groups. The negative results may be related to the small sample size and the length of observation as efficacy may take longer to demonstrate for rehabilitation therapy. Total exercise time, time from the start of exercise to the occurrence of ST depression 1 mm, time from the start of exercise to the onset of angina at 3 months after rehabilitation therapy were noticeably lengthened compared with controls. We found that ST segment moved lower during peak exercise and the maximum exercise tolerance increased significantly. The number of anginal episodes was also markedly lower in the rehabilitation training group, indicating that rehabilitation training could improve the exercise capacity of patients with coronary artery disease and blood circulation in the myocardium. Individualized exercise through prescribed exercise type, intensity, exercise interval and frequency can tailor to the needs of individuals and thus better promote cardiac function and prevent the progression of coronary heart disease[17]–[19]. One limitation of the current study is the low number of study subjects, which may limit the generalizability of our findings. In addition, our patients were recruited from a tertiary hospital setting and the findings may not be applicable to those patients who are seen in primary care settings. We are currently carrying out further study on the effect of rehabilitation training on exercise tolerance after PCI in a larger patient population from both tertiary and primary care settings.
